# Prediction of neonatal outcomes using gestational age vs ACOG definitions of maternal disease severity in hypertensive disorders of pregnancy

**DOI:** 10.1007/s00404-024-07684-y

**Published:** 2024-08-16

**Authors:** Isabella Hauptman, Kevin S. Gill, Tiffany Lim, Wendy J. Mack, Melissa L. Wilson

**Affiliations:** Keck School of Medicine, Department of Population and Public Health Sciences, University of Southern California, Los Angeles, USA

**Keywords:** Area under the curve, Case-only study, Hypertensive disorders of pregnancy

## Abstract

**Purpose:**

Hypertensive disorders of pregnancy cause significant neonatal complications. Disease severity is often used to predict neonatal outcomes, however gestational age (GA) at delivery may be a better predictor. We aimed to assess whether disease severity or GA was more predictive of adverse neonatal outcomes.

**Methods:**

We included 165 participants with confirmed HELLP syndrome or severe preeclampsia (sPE). Two predictive models were constructed to assess the ability of disease severity compared to GA to predict a composite adverse neonatal outcome. The composite outcome included low birth weight, SGA, IUGR, Apgar score, and neonatal death.

**Results:**

Using severity as a predictor of binary neonatal outcome had an AUC of 0.73 (0.65–0.81), with a sensitivity (SE) of 70.3% and a specificity (SP) of 64.4%. For GA, we observed an AUC of 0.82 (0.75–0.89), with a SE of 75.7% and a SP of 76.7%.

**Conclusion:**

For the composite neonatal outcome, GA was a better predictor than ACOG diagnosis (severity). This observation underscores the need for further research to validate these findings in larger cohorts and to determine their applicability to maternal outcomes.

## What does this study add to the clinical work

**Table:** 

We found that models based on GA are better predictors of a wide range of adverse neonatal outcomes, including low birth weight, SGA, IUGR, Apgar score, and neonatal death; when compared to ACOG definitions of maternal disease severity in hypertensive disorders of pregnancy. The study contributes to the ongoing discourse surrounding the clinical utility of diagnostic criteria in predicting adverse neonatal events and prompts a reevaluation of their role in guiding clinical decisions.	
These predictive models serve as another tool in clinical settings.	
Provides more information sooner to the clinician and patient.	
Faster, more accurate diagnosis of HDP.	
Mothers can be given the proper treatment sooner or be transferred to more equipped facility.	
Reduces adverse outcomes.	

## Introduction

Hypertensive Disorders of Pregnancy (HDP) are serious gestational complications that can pose a threat to both mother and child [1] and are leading causes of maternal and neonatal mortality in low-and middle-income countries (LMICs) [2]. Severe Preeclampsia (sPE) and Hemolysis, Elevated Liver Enzymes, and Low Platelet Count (HELLP) syndrome are at the severe end of the HDP spectrum and are associated with worse outcomes. While PE and HELLP syndrome are generally considered to fall under the HDP umbrella, recent findings suggest sPE and HELLP syndrome may be independent conditions that arise from a materno-fetal imbalance [3]. Preeclampsia occurs in 3–6% of pregnancies while HELLP syndrome occurs in 0.5–0.9% of pregnancies and in 20–25% of preeclamptic pregnancies [3]. Most neonates born to a mother with HELLP syndrome or sPE require extended hospitalization in neonatal intensive care units [3].

Clinically, obstetricians often use disease severity (mild vs severe, Type 1 vs Type 2) as a predictor for adverse outcomes, though studies have suggested that these classifications do not reliably associate with risk, as they tend to underestimate the impact of HDP at term [4]. GA at delivery (< 34 weeks vs ≥ 34 weeks) has also been suggested as a predictor of adverse outcomes, but it is unclear whether severity definitions that rely on blood pressure, proteinuria, and organ dysfunction criteria are preferable to the use of GA to predict poor outcomes [5]. Thus, it would be helpful to have a reliable way of predicting adverse outcomes other than to rely on diagnostic criteria, as these have historically changed over time [6].

Kleuskens et al. performed a systematic review of prediction models for preeclampsia, HELLP syndrome, and fetal-growth restriction, and determined all 41 models to be of low methodological quality alongside a lack of external validation [7]. Ngwenya et al. emphasized the reduced applicability and accuracy of predictive models developed using data from high-income countries toward populations in LMICs due to the large discrepancy of resources between these settings [8]. To address this issue, the mini Preeclampsia Integrated Estimate of RiSk (miniPIERS) model was developed for LMICs juxtaposing the fullPIERS model, which was developed to assess adverse maternal and neonatal outcomes in high-income countries.

Other diverse approaches have been explored for constructing predictive models for HDP-related outcomes. Morris et al. [9] centered their efforts on serum biomarker Pregnancy-Associated Plasma Protein A (PAPP-A), while Cohen et al. [10] evaluated combinations of PAPP-A, free β human Chorionic Gonadotropin (βhCG), and maternal serum Alpha-FetoProtein (msAFP) serum biomarkers to predict adverse pregnancy outcomes. However, Morris et al. [9] had poor predictive values as biomarker studies have not shown consistent promise in this population. Lafalla et al. [11] investigated a composite model integrating thrombophilia, antithrombotic drugs, and maternal––fetal characteristics, offering a predictive model for placenta-mediated pregnancy complications. Escobar et al. [12] developed a predictive model using electronic medical records to mitigate morbidity and mortality risks during childbirth in real-time. In a quantitative approach, Schwartz et al. [13] harnessed sonographic measurements of fetuses to prognosticate small-for-gestational-age (SGA) and preeclampsia (PE); however, these models showed moderate predictive capability (AUC: 0.7).

While prediction models have been developed to predict HDP [14], recurrent HDP [15] and adverse neonatal outcomes [12] such models have not yet been universally adopted, even when predictive ability is moderate to good. Khosla et al. recently examined the cost effectiveness of adopting the sFLT1/PlGF ratio as a predictor for HDP and found that, due to the decreased need for hospitalization among suspected cases, payors may save between $771 to $1330 per patient at 2020 prices [16].

The diagnostic criteria for PE were updated by the American College of Obstetricians and Gynecologists (ACOG) in 2020 and The International Society for the Study of Hypertension in Pregnancy (ISSHP) in 2014 to include hypertension in the absence of proteinuria if there is also evidence of systemic dysfunction [17]. It remains unclear if these definitions are predictive of neonatal outcomes or if other factors (e.g., gestational age at delivery) are equally or even more predictive of adverse outcomes [18, 19]. Gestational age (GA) is an established risk factor for poor neonatal and maternal outcomes and the risk of adverse outcomes is negatively associated with increasing GA up to 40 weeks [20, 21].

We aimed to investigate the clinical utility of ACOG-defined diagnosis for HELLP syndrome and sPE in predicting neonatal outcomes by examining whether the clinical diagnosis of HELLP syndrome vs sPE is more predictive of neonatal outcomes than GA alone.

## Methods

*Study population:* The study sample (*n* = 165) consisted of women with self-identified HELLP syndrome who were recruited online from two separate websites (www.hellpsyndromesociety.org and https://www.facebook.com/pages/Hellp-Syndrome-Research-at-USC/163745723652843). All women self-reported a history of HELLP syndrome. Women completed a standardized risk factor questionnaire, which included questions about their medical history, reproductive and sexual history, family history, and the affected pregnancy. Medical records were requested from the delivery hospital and the obstetrician from all cases to confirm the diagnosis. Diagnoses which could not be confirmed as HELLP syndrome were classified as sPE. A standardized data abstraction form was used to abstract the records, which included information about prenatal visits, comorbidities, obstetric history, and delivery. Missing covariate data from case abstractions were not imputed. The absence of neonatal death was confirmed through chart review.

*Exposure definition:* Participants were classified as having HELLP syndrome if medical records confirmed the following criteria: hemolysis (schistocytes, burr cells, LDH > 600, or bilirubin > 1.2), elevated liver enzymes (AST > 70 and/or ALT > 70), and low platelets (platelets < 100 K). Women meeting two of the three criteria were classified as having sPE. Women with significant hypertension (≥ 160/110 mmHg on two occasions, at least 6 h apart) and proteinuria (500 mg/dL/24 h or + 3 dipstick on two occasions at least 6 h apart) were also classified as having sPE, with or without one of the above criteria [22]. Early delivery was defined as delivery at a GA of 34 weeks or earlier.

Due to incomplete medical records (obstetric only, prenatal only, or neither), some participants are missing on variables obtained only from medical records. To demonstrate missingness, we included the sample sizes for each group for descriptive variables (Tables 1 and 2). We did not perform imputation since the variables included in the models (GA/ACOG definition, blood pressure, edema, and infant sex) were generally available from the medical records or the accompanying questionnaire.

**Table 1:** Demographic and clinical characteristics of the study population categorized by ACOG diagnosis

Variable^a^	N	sPE	N	HELLP syndrome	*p* value^b^
*Demographic characteristics*					
Maternal age, years	97	30.8 (± 3.7)	68	30.1 (± 4.0)	0.21
White	90	89 (98.9%)	64	63 (98.4%)	> 0.99
Nulliparity	96	84 (87.5%)	66	59 (89.4%)	0.71
*Medical history*					
History of hypertension	91	11 (12.1%)	67	3 (4.5%)	0.10
History of diabetes	94	6 (6.4%)	66	4 (6.1%)	> 0.99
Pre-pregnancy weight, lbs	90	147.3 (± 29.9)	57	148.1 (± 38.1)	0.47
Maximum systolic blood pressure (mmHg)	93	161.5 (± 24.3)	67	162.6 (± 23.0)	0.77
Maximum diastolic blood pressure (mmHg)	93	98.7 (± 12.0)	67	99.2 (± 15.0)	0.90
*Laboratory measurements*					
Maximum LDH (units/L)	40	509.3 (± 411.7)	50	2155.7 (± 3337.9)	< 0.01
Maximum bilirubin (mg/dL)	56	0.7 (± 0.4)	56	4.1 (± 12.5)	< 0.01
Maximum AST (units/L)	87	290.5 (± 394.4)	67	625.8 (± 905.9)	< 0.01
Maximum ALT (units/L)	78	262.5 (± 341.5)	66	580.5 (± 1316.0)	< 0.01
Maximum creatinine (mg/dL)	74	3.6 (± 15.1)	59	5.4 (± 19.2)	0.78
Minimum platelet count (× 10^9^/ L)	89	101.9 (± 68.1)	68	47.5 (± 20.2)	< 0.01
*Perinatal events*					
Delivery type	87		62		0.17
Vaginal (spontaneous)		20 (22.9%)		8 (12.9%)	
Cesarean section		63 (72.4%)		53 (85.5%)	
Vacuum-assisted		4 (4.6%)		1 (1.6%)	
Maternal hemorrhage	90	4 (4.4%)	65	5 (7.7%)	0.49
Birthweight (g)	82	1989.9 (± 977.9)	61	1925.8 (± 945.4)	0.56
Small for gestational age	80	20 (25.0%)	59	14 (23.7%)	0.86
IUGR	92	12 (13.0%)	64	10 (15.6%)	0.65
Apgar score	77		57		0.08
0–4		20 (20.6%)		7 (10.3%)	
5–10		77 (79.4%)		61 (89.7%)	
Neonatal death	97	15 (15.5%)	68	4 (5.9%)	0.06

Lactate Dehydrogenase (LDH); Aspartate Aminotransferase (AST); Alanine Aminotransferase (ALT); Intrauterine Growth Restriction (IUGR)

^a^Continuous variables presented as mean (± standard deviation) and categorical variables presented as frequencies (%)

^b^P values obtained by t-test or Wilcoxon rank sum for continuous variables as appropriate and by Pearson’s Chi-square test or Fisher’s Exact test for categorical variables as appropriate

**Table 2:** Demographic and clinical characteristics of the study population categorized by gestational age at delivery

Variable^a^	N	Gestational age > 34 weeks	N	Gestational age ≤ 34 weeks	*p* value^b^
Demographic characteristics					
Maternal age, years	72	30.8 (± 3.7)	93	30.3 (± 4.0)	0.31
White	63	63 (100%)	91	89 (97.8%)	0.51
Nulliparity	69	62 (89.9%)	93	81 (87.1%)	0.59
Medical history					
History of Hypertension	67	5 (7.5%)	91	9 (9.8%)	0.60
History of diabetes	70	3 (4.3%)	90	7 (7.8%)	0.52
Pre-pregnancy weight, lbs	63	146.6 (± 31.9)	84	148.4 (± 34.30)	0.90
Maximum systolic blood pressure (mmHg)	70	157.3 (± 25.5)	90	165.5 (± 21.7)	0.03
Maximum diastolic blood pressure (mmHg)	70	97.7 (± 14.0)	90	99.8 (± 2.8)	0.39
Laboratory measurements					
Maximum LDH (units/L)	35	867.5 (± 994.4)	55	1778.0 (± 3224.4)	0.09
Maximum Bilirubin (mg/dL)	52	1.8 (± 2.5)	60	2.4 (± 12.0)	0.74
Maximum AST (units/L)	67	425.1 (± 620.8)	87	445.1 (± 734.0)	0.83
Maximum ALT (units/L)	65	354.4 (± 533.6)	79	452.6 (± 1169.0)	0.77
Maximum creatinine (mg/dL)	58	2.6 (± 9.7)	75	5.9 (± 20.9)	0.43
Minimum platelet count (× 10^9^/ L)	70	84.2 (± 71.2)	87	73.6 (± 47.7)	0.77
*Perinatal events*					
Delivery type	64		85		0.008
Vaginal (spontaneous)		15 (23.4%)		13 (15.3%)	
Cesarean section		44 (68.8%)		72 (84.7%)	
Vacuum-assisted		5 (7.8%)		0 (0%)	
Maternal hemorrhage	66	6 (9.1%)	89	3 (3.4%)	0.17
Birthweight (g)	63	2769.5 (± 699.7)	80	1327.1 (± 590.5)	< 0.01
Small for gestational age	59	5 (8.5%)	80	29 (36.3%)	< 0.01
IUGR	66	3 (4.5%)	90	19 (21.1%)	< 0.01
Apgar score	72		93		0.11
0–4		8 (11.1%)		19 (20.4%)	
5–10		64 (88.9%)		74 (79.6%)	
Neonatal death	72	3 (4.2%)	93	16 (17.2%)	< 0.01

*LDH* lactate dehydrogenase; *AST* aspartate aminotransferase; *ALT* alanine aminotransferase; *IUGR* intrauterine growth restriction

^a^Continuous variables presented as mean (± standard deviation) and categorical variables presented as frequencies (%)

^b^P-values obtained by t test or Wilcoxon rank sum for continuous variables as appropriate and by Pearson’s Chi-square test or Fisher’s Exact test for categorical variables as appropriate

*Outcome definition:* The following neonatal outcomes were included as a binary composite measure that was defined by the presence of any one of these factors: SGA (birth weight < 10th percentile per gestational week and gender) as defined by Olson et al. [23], a 1-min Apgar score of ≤ 4, intrauterine growth restriction (IUGR) documented by ultrasound, very low birthweight (defined as less than 1500 g), or neonatal death.

**Fig. 1:**
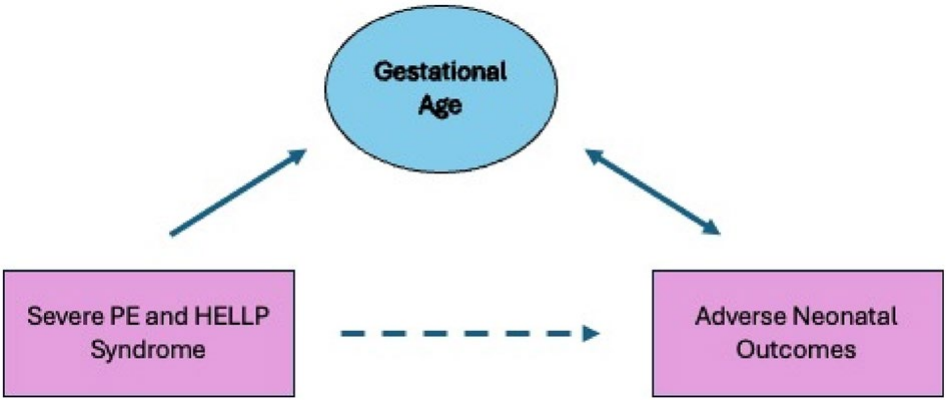
When examining the between diagnosis and adverse neonatal outcomes, it is not appropriate to consider gestational age as a covariate, since it is a collider variable. Specifically, the diagnosis of severe preeclampsia or HELLP leads to an earlier age at delivery and is also strongly associated with adverse neonatal outcomes, as shown in the figure. The inclusion of a collider variable results in a biased estimate

*Statistical methods:* To investigate the clinical utility of the ACOG diagnoses for HELLP syndrome and sPE compared to early deliveries in predicting neonatal outcomes, we developed 2 predictive models developed for each main exposure of interest (ACOG diagnosis/severity and delivery ≤ 34 weeks). The following independent candidate covariates were considered for possible inclusion in the predictive models: maternal age at pregnancy confirmation, pre-pregnancy weight, GA at first prenatal care visit, maternal history of asthma, diabetes, chronic hypertension, delivery type, headache, epigastric pain, edema, nausea, visual symptoms, maximum LDH, bilirubin, AST, ALT, creatinine levels, minimum platelet levels, child birth weight, maximum systolic blood pressure, maximum diastolic blood pressure, white blood cell count, nulliparity, maternal hemorrhage, blood type, eclampsia, and placental abruption. We did not include GA in our model using ACOG diagnosis as it is a collider variable in the relationship between HDP and adverse neonatal outcomes (Fig. 1). Instead, we used GA (≤ 34 weeks vs > 34 weeks) as a predictor of adverse neonatal outcomes, independent of diagnosis.

Demographic and clinical characteristics of the population, stratified according to ACOG diagnosis and early delivery are reported as means ± standard deviation for numeric variables and frequencies with percent for categorical variables. Statistical tests comparing sPE to HELLP and GA > 34 weeks to GA $\le$ 34 weeks were performed using *t* tests for normally distributed continuous variables, Wilcoxon rank sum tests for non-normally distributed continuous variables, Pearson’s Chi-square, and Fisher’s Exact tests.

*Predictive modeling:* Bivariate analyses were performed with logistic regression on neonatal outcome and each candidate variable. Eligible variables for the preliminary model were defined as having a bivariate *p* value of < 0.25. We then performed multivariable logistic regression for each exposure definition and tested the significance of each candidate covariate. Subsequent variables that did not meet statistical significance of ≤ 0.05 were removed from the preliminary model in order of decreasing significance. Variables with a Wald* p* value of ≤ 0.05 were maintained in the final model.

After the preliminary main effects models were finalized, the linearity of the continuous variables was assessed. Fractional polynomials were calculated from the adjusted preliminary models. Considerations were made for both one and two term power functions in comparison to linear models for each continuous variable. The greater power term was selected if *p* ≤ 0.05. To maintain consistency across models, variables for each model/outcome pair were kept consistent. Goodness of fit was determined using the Hosmer–Lemeshow (HL) test set to four groups, neither HL was statistically significant, suggesting adequate fit. We further assessed model fit by inspection of residuals and influence.

The resulting regression estimates were reported as odds ratios. The ROC curve, the area under the ROC curve (AUC), and the classification table formed at the cutpoint deemed to maximize the sensitivity and specificity of the model are reported. The maximized cutpoint was assessed by graphing sensitivity and specificity vs probability cutoff to determine where both specificity and sensitivity were maximal. We compared the AUC between each model to determine which model had better discrimination. If the models were statistically significantly different (*p* ≤ 0.05), the model with higher AUC was identified as the preferred model. All analyses were conducted using Stata 16 (Statacorp, College Station, TX) [24].

This study was conceived as an exploratory study and thus no a priori power calculation was made. Post hoc, we calculated the precision with which we could estimate the two-sided 95% confidence interval (CI) assuming an AUC of 0.70. For the model using the ACOG diagnostic criteria (Model 1) were able to estimate the AUC to be between 0.53 and 0.87 with 95% confidence with the available 68 subjects from the HELLP population and 97 subjects from the sPE population. The model using GA as a predictor (Model 2) would be able to estimate an AUC of 0.70 to be between 0.54 and 0.86 with 95% confidence with 93 subjects from the early GA group and 72 subjects from the later GA group. Precision for confidence intervals was calculated using PASS 14 [25].

## Results

The study population consisted of 165 individuals, of which 68 (41%) were confirmed to have HELLP syndrome and 97 (59%) were confirmed cases of sPE (Table 1). In addition, 93 (56%) individuals gave birth at or below a GA of 34 weeks and 72 (44%) gave birth after 34 weeks (Table 2**).** The total sample size in each multivariable prediction model varies based on the available data for each covariate.

No statistically significant difference was observed between mothers diagnosed with sPE and HELLP syndrome with regard to: maximum creatinine levels (mg/dL) (*p* = 0.78), delivery type (*p* = 0.16), maternal hemorrhage (*p* = 0.39), birthweight (g) (*p* = 0.56), SGA (*p* = 0.86), IUGR (*p* = 0.65), Apgar Score (*p* = 0.08), neonatal death (*p* = 0.06), prior history of hypertension (p = 0.10), prior history of diabetes (*p* = 0.93), mean pre-pregnancy weight (lbs) (*p* = 0.47), maximum systolic blood pressure (mmHg) (*p* = 0.77), and maximum diastolic blood pressure (mmHg) (*p* = 0.90) (Table 1). Statistically significant differences were observed between mothers diagnosed with sPE and HELLP syndrome with the following variables related to laboratory measurements: higher maximum LDH (units/L) in the HELLP group (2155.7 ± 3337.9) compared to the sPE group (509.3 ± 411.7) (*p* < 0.001), higher maximum bilirubin (mg/dL) in the HELLP group (4.1 ± 12.5) compared to the sPE group (0.7 ± 0.4) (*p* < 0.001), higher maximum AST (units/L) in the HELLP group (625.8 ± 905.9) compared to the sPE group (290.5 ± 394.4) (*p* < 0.001), higher maximum ALT (units/L) in the HELLP group (580.5 ± 1316.0) compared to the sPE group (262.5 ± 341.5) (*p* = 0.001), and minimum platelet count (× 10^9^/ L) in the HELLP group (47.5 ± 20.2) compared to the sPE group (101.9 ± 68.1) (*p* < 0.001) (Table 1).

No statistically significant difference was observed between mothers who gave birth at or below 34 weeks and those who gave birth later than 34 weeks with the following variables related to medical history: prior history of hypertension (*p* = 0.60), prior history of diabetes (*p* = 0.36), mean pre-pregnancy weight (lbs) (*p* = 0.90), and maximum diastolic blood pressure (mmHg) (*p* = 0.39) (Table 2). However, a statistically significant difference was noted for maximum systolic blood pressure (mmHg), with a higher systolic blood pressure in the earlier delivery group (165.5 ± 21.7) compared to the later delivery group (157.3 ± 25.5) (*p* = 0.030) (Table 2). No statistically significant difference was observed between the two delivery groups among variables related to laboratory measurements: maximum LDH (units/L) (p = 0.09), maximum bilirubin (mg/dL) (*p* = 0.74), maximum AST (units/L) (*p* = 0.83), maximum ALT (units/L)(p = 0.70), maximum creatinine levels (mg/dL) (*p* = 0.43), and minimum platelet count (× 10^9^/ L) (*p* = 0.39) (Table 2). Statistically significant differences were detected for: delivery type, with less vaginal and vacuum assisted deliveries and more Cesarean Section deliveries in the early delivery group compared to the later delivery group (*p* = 0.010), lower birth weight (g) in the earlier delivery group (1327.1 ± 590.5) compared to the later delivery group (2769.5 ± 699.7) (*p* < 0.001), greater frequency of SGA in the earlier delivery group (36.3%) compared to the later delivery group (8.5%) (*p* < 0.001), greater frequency of IUGR in the earlier delivery group (21.1%) compared to the later delivery group (4.5%) (*p* = 0.003), and greater frequency of neonatal death in the earlier delivery group (17.2%) compared to the later delivery group (4.2%) (*p* = 0.009) (Table 2). No significant differences were observed for maternal hemorrhage (*p* = 0.17) or Apgar Score (*p* = 0.11) (Table 2).

**Fig. 2:**
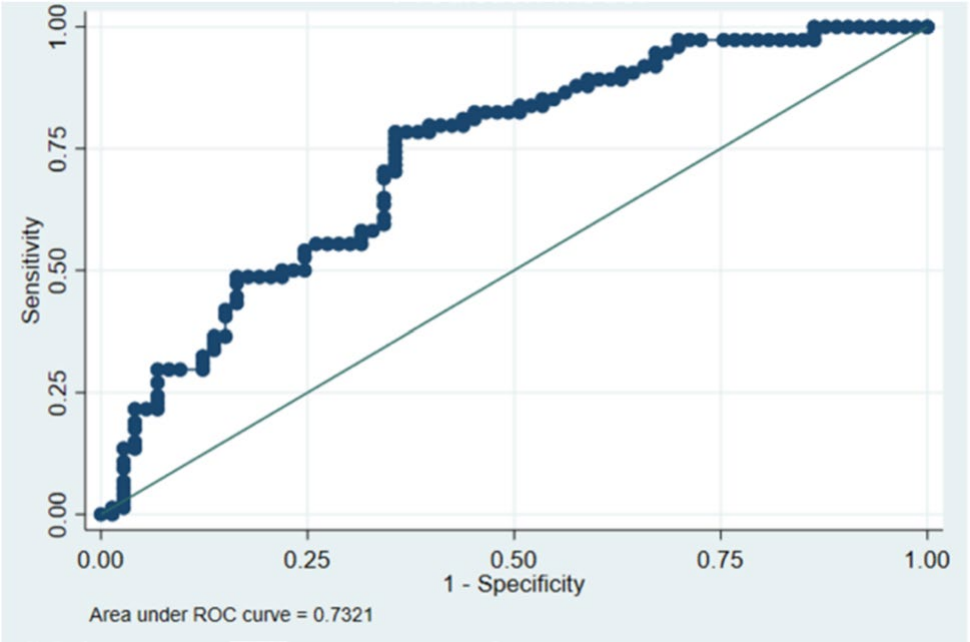
Area under ROC curve (Model A)

Model A—Composite Neonatal Outcome Including Low Birthweight, SGA, IUGR, Apgar Score, and Neonatal Death using ACOG definition**:** We observed a nonsignificant reduction in neonatal death (OR = 0.52, 95% CI 0.25–1.08, *p* = 0.08, Table 3) for those with HELLP syndrome compared to those with sPE. Maximum systolic blood pressure was significantly higher in those with adverse neonatal outcomes (OR = 1.03, 95% CI 1.01–1.04, *p* = 0.001 Table 3). In addition, those with edema were significantly more likely to have an adverse outcome (OR = 2.58, 95% CI 1.25–5.33, *p* = 0.010 Table 3) along with male neonates (OR = 2.09, 95% CI 1.01–4.53, *p* = 0.048 Table 3). The AUC for this model was estimated to be 0.73 (95% CI 0.65–0.81) (Table 3, Fig. 2**)**. As parameterized, the model had a sensitivity of 70.3% and a specificity of 64.4% with a correct classification rate of 67.4% (Table 3**)**.

**Table 3:** Predictive model for adverse neonatal events using ACOG diagnostic criteria (Model A)

Variable	Odds ratio	95% confidence interval		*p* value
ACOG definition				
Severe PE	Ref	Ref	Ref	Ref
HELLP	0.52	0.25	1.08	0.08
Maximum systolic blood pressure (mmHg)	1.03	1.01	1.04	< 0.01
Edema	2.58	1.25	5.33	0.01
Male infant	2.09	1.01	4.35	0.05
Sensitivity	70.3%			
Specificity	64.4%			
AUC	0.73 (0.65, 0.81)			

*n* = 147; LR χ^2^ (4) = 23.03; *p* = 0.0001; Hosmer–Lemeshow χ^2^ (2) = 0.83, *p* = 0.66

**Fig. 3:**
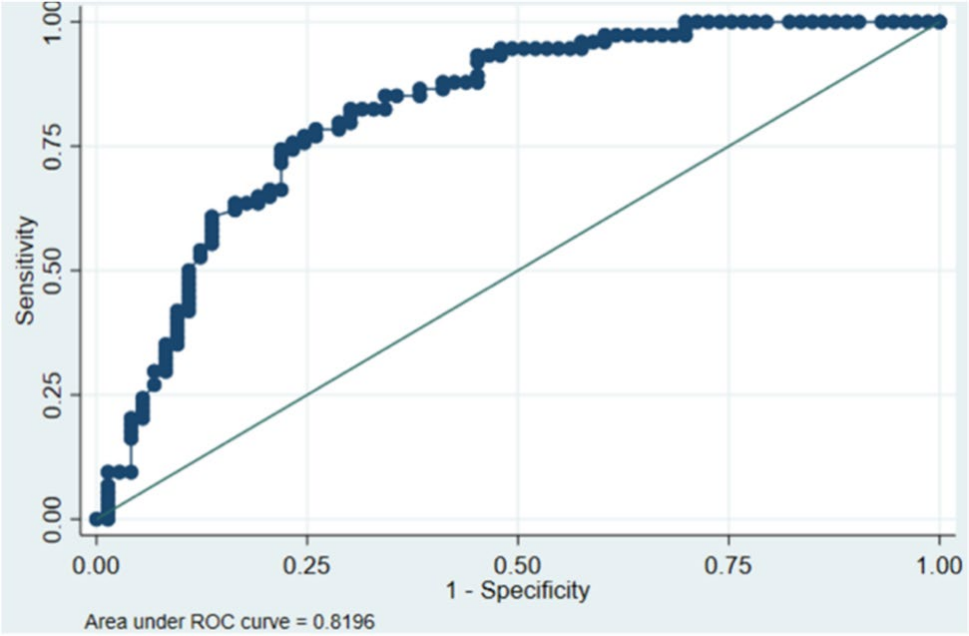
Area under ROC curve (Model B)

Model B—Composite Neonatal Outcome Including Low Birthweight, SGA, IUGR, Apgar Score, and Neonatal Death using early delivery (GA): Using early delivery to predict risk of neonatal outcomes, we found a significant increase in the odds of an adverse outcome associated with delivering at ≤ 34 weeks compared to those who delivered at > 34 weeks (OR = 8.53, 95% CI 3.70–18.86,* p* < 0.001, Table 4). As before, maximum systolic blood pressure is significantly higher in those with adverse outcomes, independent of GA (OR = 1.03, 95% CI 1.01–1.04, *p* = 0.008 Table 4). In addition, those with edema were significantly more likely to have a neonatal complication, independent of GA, gender, or blood pressure (OR = 2.53, 95% CI 1.14–5.65, *p* = 0.023 Table 4). However, male neonates were not at significantly increased risk in this model (OR = 1.47, 95% CI 0.66–3.26, *p* = 0.35 Table 4). When examining predictive capacity of the model, we estimated the AUC to be 0.82 (95% CI 0.75–0.89) (Table 4, Fig. 3). As parameterized, the model had a sensitivity of 75.7% and a specificity of 76.7% with a correct classification rate of 76.2% (Table 4). A comparison of the AUC between Models A and B suggests that they are statistically significantly different (*p* = 0.031), with the GA-based model showing better predictive ability than the model based on ACOG definitions.

**Table 4:** Predictive model for adverse neonatal events using gestational age (Model B)

Variable	Odds ratio	95% confidence interval		*p* value
Gestational age				
> 34 weeks	Ref	Ref	Ref	Ref
≤34 weeks	8.35	3.70	18.86	< 0.01
Maximum systolic blood pressure (mmHg)	1.03	1.01	1.04	< 0.01
Edema	2.53	1.14	5.65	0.02
Male	1.47	0.66	3.26	0.35
Sensitivity	75.7%			
Specificity	76.7%			
AUC	0.82 (0.75, 0.89)			

*n* = 147 LR χ^2^ (4) = 50.07; *p* < 0.001 Hosmer–Lemeshow χ^2^ (2) = 0.83, *p* = 0.66

## Discussion

We developed two predictive models to investigate the added clinical utility of the ACOG diagnosis for sPE vs HELLP syndrome in predicting adverse neonatal outcomes. The GA-based exposure model performed better than the ACOG-defined exposure model in providing additional predictive utility for adverse neonatal outcomes. Unexpectedly, we found that HELLP syndrome, compared to sPE, was associated with a decreased risk of an adverse outcome, though this difference did not reach significance. Delivering at or below 34 weeks of gestation was a significant predictor of adverse neonatal outcomes. In addition, maximum systolic blood pressure was associated with a significant increase in risk of an adverse outcome.

These findings are not consistent with prior studies that evaluated neonatal outcomes in women with sPE and HELLP syndrome. Few studies attempted to model the added predictive power of the ACOG diagnoses and instead reported on associations with various adverse neonatal outcomes between those with HELLP syndrome and sPE. Gul et al. [26] found that neonatal and perinatal mortality was significantly higher in the HELLP group vs the sPE group but did not find any significant difference between the groups with respect to IUGR and Apgar score. Controlling for GA at delivery, these differences were insignificant. Similarly, Turgut et al. [27] found that neonates born to women with HELLP syndrome had significantly lower neonatal bodyweight and higher neonatal mortality compared to women with sPE. As with Gul et al. [26], neonatal mortality and morbidity were found to be mediated by GA. When stratified by GA, the association between neonatal adverse outcomes and diagnosis are attenuated and nonsignificant. Abramovici et al. [18] also found that neonates born to women with HELLP syndrome had significantly lower birth weight, earlier GA at delivery, and a higher frequency of 5 min Apgar scores less than 7 compared to neonates born to women with sPE, but the association becomes null when stratified as < 28 weeks, 29–32 weeks, and 33–36 weeks of GA. Haddad et al. [28], found no association between an increased risk in neonatal adverse outcomes among women with HELLP syndrome diagnosed at or before 28 weeks of gestation compared to women with sPE diagnosed at or before 28 weeks.

Several differences between these studies may account for the discrepancy in findings. Our study consisted of an almost entirely white study population with ready internet access. Unlike PE, which is more prevalent in Black women, HELLP syndrome is more common in white women [29]. Both Abramovici et al. [18] and Haddad et al. [28] examined HELLP syndrome and sPE in study populations that were predominantly Black. Further, the respective diagnoses of HELLP syndrome and sPE used in this study were based upon the 2020 ACOG criteria. Gul et al. [26], Turgut et al. [27], Haddad et al. [28], and Abramovici et al. [18] defined sPE using the 1996 ACOG criteria, which excluded severe gestational hypertension in the absence of proteinuria with other clinical features. Historically, studies of PE regularly adjusted for GA. However, GA should not be included in models examining risk factors for neonatal outcomes as GA is likely to be a collider, not a confounder [29] (Fig. 3). Similarly, collider- stratification bias can result when conditioning on a shared effect, such as GA, which affects both neonatal outcomes and PE. Adjusting for a collider can lead to substantial negative bias [30, 31]. Thus, we would expect to see bias toward the null when stratifying by GA, which was observed by Gul et al. [26], Turgut et al. [27], Haddad et al. [28], and Abramovici et al. [18]. By not adjusting for GA in our ACOG-defined models, our results would not have experienced this attenuation.

In this study, we found that the ACOG diagnoses models were statistically less predictive of neonatal outcomes compared to models using GA ($\le$ 34 weeks vs > 34 weeks). Specifically, our GA model was significantly better at predicting the composite neonatal adverse events of low birthweight, SGA, IUGR, 1 min Apgar Score of 4 or less, or neonatal death (Model B). These findings suggest that prediction of neonatal morbidity and mortality is improved using GA at delivery rather than the presence of sPE vs HELLP syndrome.

These results are supported by previous findings. Kinay et al. [32] examined maternal characteristics and perinatal outcomes between women with sPE and HELLP syndrome in two separate groups: women who gave birth at or less than 34 weeks gestation and more than 34 weeks gestation. They did not find a statistically significant difference in perinatal outcomes between patients with sPE and HELLP syndrome in either GA category, suggesting that ACOG diagnoses may be a suboptimal predictor of neonatal outcomes [32]. A study by Menzies et al. [33] examined the predictive power of PE severity in an international cohort. The study found little evidence that sPE predicted adverse neonatal outcomes, with the exception of diastolic blood pressure greater than 110 mmHg and suspected placental abruption [33].

Our findings support the conclusion that the ACOG diagnosis of sPE does not predict adverse neonatal outcomes as well as GA. Although HELLP syndrome and sPE have defined diagnostic criteria, the clinical utility of the diagnoses for predicting adverse neonatal events are in question. The rigidity of the definitions, the dynamic nature of delivery, and varying interventions employed to manage symptoms can all impact the ultimate diagnosis. Exactly how much overlap there is between sPE and HELLP syndrome is an area of active research, with some studies suggesting substantial overlap [34, 35] and others suggestive of differing underlying pathophysiology [36]. As a result, misclassification along the spectrum of HDP is likely, potentially explaining the limited predictability of the ACOG definitions. In contrast to the myriad difficulties of diagnosing a dynamic condition, GA has less potential for misclassification and thus may be a better predictor of neonatal outcomes.

This study has several strengths. Medical laboratory data were available through medical record abstraction, allowing us to verify the diagnoses as well as evaluate specific laboratory values as covariates. In addition, the cohort consists of severe-spectrum HDP, a population more likely to experience neonatal complications and therefore, the ability to predict adverse outcomes may be most relevant.

This study also has several limitations. First, the study population is small (*n* = 165), leading to limited power to detect differences between the AUC curves. However, our study population represents severe-spectrum HDP, which impacts < 2% of all pregnancies [37] and thus, large numbers of cases are difficult to obtain [38]. Second, participants were self-identified and opted into the study from online resources. Therefore, it is unknown how many women with HELLP syndrome or sPE accessed the websites and thus, we are unable to calculate participation rates or evaluate selection bias. Third, the potential for misclassification of HELLP syndrome vs sPE is not insignificant, since diagnosis of HELLP syndrome requires complete blood and chemistry panels and timing of the assays can determine whether a diagnosis of HELLP syndrome is made. If these tests were not performed or did not meet the cut points set for a diagnosis of HELLP syndrome, the participants were classified as having sPE; potentially leading to the underreporting of HELLP syndrome in this population. If indeed some HELLP syndrome cases had been misclassified as sPE, any observed differences between these groups would be attenuated. Since we did find significant differences between groups with respect to several factors, we do not expect that misclassification can entirely explain our results. Last, due to lack of clarity regarding neonatal death vs stillbirth, we were unable to include stillbirth in our outcome definition.

The results of our exploratory study support the use of GA as a predictor of adverse neonatal outcomes over the diagnosis of HELLP syndrome vs sPE. Specifically, we observed that the model developed with GA as a predictor improved the predictive ability for adverse neonatal outcomes compared to the model developed with diagnosis of HELLP syndrome and sPE. Further research is suggested to examine the clinical utility of these diagnoses with respect to maternal outcomes and to confirm our findings in a larger study.

## Data Availability

The data used in this analysis is available upon request to the corresponding author.
